# Oxygen-generating Microparticles Enhance Viability and Functionality of Human Pluripotent Stem Cell-derived Cardiomyocytes for Myocardial Infarction Therapy

**DOI:** 10.1007/s12015-026-11163-z

**Published:** 2026-05-27

**Authors:** Xingyu He, Suchandrima Dutta, Darshini Desai, Sheng Zhong, William Liu, Sophie Chen, Wei Huang, Waqas Ahmad, Jialiang Liang, Yigang Wang

**Affiliations:** 1 Department of Pathology and Laboratory Medicine, College of Medicine, University of Cincinnati, 231 Albert Sabin Way, Cincinnati, OH 45267 USA; 2 Department of Pharmacology, Physiology and Neurobiology, College of Medicine, University of Cincinnati, Cincinnati, OH 45267 USA; 3 Department of Cancer Biology, College of Medicine, University of Cincinnati, Cincinnati, OH 45267 USA; 4 Division of Pharmaceutical Sciences, James L. Winkle College of Pharmacy, University of Cincinnati, Cincinnati, OH 45229 USA; 5 Department of Internal Medicine, College of Medicine, University of Cincinnati, Cincinnati, OH 45267 USA

**Keywords:** Oxygen-generating microparticles, HiPSC-derived cardiomyocytes, Engineered heart tissue, Hypoxia, Myocardial infarction

## Abstract

**Background:**

Human induced pluripotent stem cell-derived cardiomyocytes (hiPSC-CMs) represent a promising therapy for myocardial infarction (MI), but their survival is severely limited by the hypoxic infarct environment. The optimal oxygen levels required to maintain the viability and functionality of hiPSC-CMs remain poorly defined. This study aimed to develop a controlled oxygen-delivery system to support engineered heart tissue (EHT) for cardiac regeneration.

**Methods:**

Oxygen-generating particles (OGPs) were engineered using peroxide (sodium percarbonate) and antioxidant (β-carotene (βCAR)) components encapsulated in PLGA microparticles. The effects of OGPs on hiPSC-CMs were evaluated through oxidative stress assays, cell viability analysis, and contractility measurements. RNA-seq was performed to investigate gene expression changes in hiPSC-CMs in response to OGPs and hypoxic stress. hiPSC-CMs combined with OGPs were encapsulated in a 3D hydrogel to generate oxygen-releasing engineered heart tissue (OR-EHT), which was implanted into infarcted hearts of immunodeficient mice. Cardiac function was assessed by echocardiography, and cell engraftment was evaluated using immunostaining.

**Results:**

OGPs provided controlled oxygen release for up to 22 days. Inclusion of βCAR minimized OGP-induced oxidative stress, preserved mitochondrial membrane potential, and maintained cell viability. OGP treatment enhanced calcium signaling and contractility in hiPSC-CMs. Transcriptomic analysis revealed that genes associated with CM maturation and contractile function were upregulated following OGP pretreatment. In addition, OGP pretreatment significantly reduced HIF-1α expression, decreased mitochondrial fragmentation, and improved survival. RNA-seq further demonstrated activation of oxygen-responsive metabolic pathways that facilitated cellular adaptation to hypoxic stress. In vivo, OR-EHT implantation for 6 weeks improved cardiac function, increased ejection fraction, reduced ventricular remodeling, and decreased infarct size compared with EHT without OGPs. Moreover, OGP incorporation significantly enhanced engraftment and survival of transplanted hiPSC-CMs and supported features consistent with early structural integration with host myocardium.

**Conclusion:**

OGP-mediated oxygen delivery offers a promising strategy for oxidative preconditioning and significantly improves the regenerative efficacy of hiPSC-CM-based cardiac therapies.

**Supplementary Information:**

The online version contains supplementary material available at 10.1007/s12015-026-11163-z.

## Introduction

Myocardial infarction (MI) causes extensive cardiomyocyte (CM) death and leads to replacement of injured myocardium with non-contractile fibrotic scar, driving adverse ventricular remodeling and cardiac dysfunction [1]. The adult mammalian heart has limited regenerative capacity such that the necrotic myocardium is not replaced after ischemic injury. Although contemporary revascularization strategies can reduce ischemic burden and preserve viable tissue, they do not regenerate new contractile myocardium once CMs have been lost [2]. These limitations have driven major interest in regenerative approaches designed to restore remuscularization of the infarcted heart.

Advances in stem cell biology and directed differentiation have positioned human induced pluripotent stem cell-derived cardiomyocytes (hiPSC-CMs) as a promising therapeutic platform for cardiac repair. hiPSC-CMs offer an abundant and patient-specific source of CMs and have demonstrated the capacity to re-muscularize infarcted myocardium in preclinical models [3]. In nonhuman primates, transplantation of human embryonic stem cell-derived CMs has been shown to partially restore myocardial tissue, establish electromechanical coupling with host myocardium, and improve ventricular function [4, 5]. However, these studies also highlight persistent translational barriers, including incomplete maturation, limited long-term graft efficiency, and post-transplant arrhythmogenic risk [4, 5].

A major determinant of poor graft survival is the hostile microenvironment of the infarcted heart, where reduced perfusion, low oxygen tension, oxidative stress, and inflammatory signaling severely impair donor-cell viability and function [6]. These stressors also disrupt mitochondrial integrity and metabolic homeostasis in transplanted CMs, further limiting early graft survival [6–8]. Among them, hypoxia plays a major role in shaping CM stress responses through stabilization of hypoxia-inducible pathways, particularly HIF-1α-dependent transcriptional programs [8, 9]. Persistent activation of the HIF-1α-LDHA (lactate dehydrogenase A) axis hinders metabolic maturation in hiPSC-CMs and maintains a glycolytic phenotype, whereas suppression of this pathway enables oxidative metabolism and functional maturation [9]. Insufficient oxygen availability in the infarcted myocardium not only impairs survival of transplanted CMs but also restricts their ability to achieve the metabolic transition required for functional integration.

To improve the local microenvironment, several strategies have been explored to elevate or stabilize oxygen availability at the injury site. Systems based on peroxide chemistry such as calcium peroxide, magnesium peroxide, and sodium percarbonate have been widely investigated for their ability to produce oxygen in situ through controlled hydrolytic decomposition [10, 11]. While such systems can increase local oxygen tension, they often exhibit limited control over oxygen release kinetics and duration [12]. To address these limitations, peroxide-based oxygen sources have been incorporated into microparticles, hydrogels, and scaffold platforms to prolong oxygen retention and enable localized delivery [13]. These approaches have shown benefits in enhancing cell survival, promoting angiogenesis, and supporting tissue repair in preclinical models of ischemic injury [11–13]. However, the generation of oxygen via peroxide decomposition is intrinsically coupled with the formation of reactive intermediates, including hydrogen peroxide, which can induce oxidative stress and negatively impact cellular viability and mitochondrial function [14, 15]. Recent designs have attempted to mitigate, this issue through catalytic components such as catalase [16, 17], or nanozyme mimetics [18], or through advanced material architectures including core–shell particles and hydrogel depots to achieve sustained and spatially controlled oxygen delivery [19]. Nevertheless, the performance of catalase and nanozyme mimetics remains highly sensitive to microenvironmental factors such as pH, temperature, and substrate availability, leading to variability in oxygen generation efficiency in vivo [12, 20]. Alternative oxygen-carrying systems such as perfluorocarbons avoid peroxide-related toxicity by physically dissolving oxygen, but are limited by low oxygen-storage capacity, short release duration, and poor integration with cellular metabolic demands [21, 22].

To address these challenges, we developed a multifunctional oxygen-generating particle (OGP) system designed to enhance oxygen availability while maintaining redox homeostasis for the engineering of hiPSC-CM-based cardiac tissue. In this system, sodium percarbonate (SPC) was encapsulated within poly(lactic-co-glycolic acid) (PLGA) microparticles in combination with the natural antioxidant β-carotene (βCAR). The incorporation of βCAR was intended to neutralize excess reactive oxygen species (ROS) generated during oxygen release, thereby mitigating oxidative stress and promoting hiPSC-CM viability. This platform was further integrated with a three-dimensional hydrogel matrix to construct engineered heart tissues (EHTs), enabling localized, sustained oxygen delivery while preserving redox balance. Finally, we demonstrated the therapeutic potential of the OGP-enabled EHTs in relevant models.

## Materials and Methods

### Preparation of OGPs

Oxygen-generating PLGA microparticles were fabricated using a modified oil-in-water (O/W) emulsion-solvent evaporation method. PLGA was dissolved in dichloromethane (DCM) at a concentration of 100 mg/mL, after which sodium percarbonate (20% w/w relative to PLGA) and βCAR (1–2% w/w relative to PLGA) were added and dispersed using probe sonication (30-s pulse in an ice bath) to ensure homogeneous suspension of oxygen-generating components. The organic phase was then emulsified into a 1% (w/v) polyvinyl alcohol (PVA) aqueous solution using high-speed homogenization at 12,000 rpm for 2 min to produce an O/W emulsion targeting a final microparticle size of 5–20 µm. After emulsification, the mixture was stirred magnetically at room temperature for 4 h to allow solvent diffusion and evaporation, leading to particle solidification. Microparticles were collected by centrifugation at 5,000 × g for 5 min, washed 3 times with sterile deionized water to remove residual PVA, and frozen at −80 °C overnight, then lyophilized for 48 h. The dry OGPs were stored at −80 °C until use.

### Oxygen-releasing Profile

The oxygen-releasing behavior of the PLGA microparticles was assessed using a Clark-type oxygen probe to quantify sustained oxygen generation (0–20 mg/L). Microparticles were suspended in PBS (pH 7.4) at 37 °C at a concentration of 1–2 mg/mL, and samples were continuously stirred to maintain homogeneity. Oxygen tension was recorded at 1-min intervals for the first hour and at 10–30 min intervals thereafter until levels stabilized. Measurements were performed in sealed chambers to prevent atmospheric oxygen exchange. Data were normalized to baseline dissolved oxygen in PBS and plotted to characterize burst release, sustained release, and total oxygen-generation capacity.

### hiPSC Culture and Differentiation

Human induced pluripotent stem cell (hiPSC) lines (ATCC-CYS0105) were maintained under standard pluripotent culture conditions in mTeSR Plus basal medium (Stemcell Technologies) and passaged at ~ 70% confluency. Six-well plates were coated with extracellular matrix by diluting 50 µL of ATCC basement membrane gel (ACS-3035) in 5 mL cold serum-free DMEM/F-12 and incubating at 37 °C for 1 h. For CM differentiation, hiPSCs were dissociated into a single-cell suspension using 0.5 mM EDTA and replated onto matrix-coated plates (either Matrigel or ATCC basement membrane gel) at ~ 70% confluency. Mesoderm induction was initiated by treating cells with 10 µM CHIR99021 in RPMI 1640 supplemented with B-27 minus insulin for 24 h, followed by 2 µM CHIR99021 for an additional 24 h. At ~ 48 h, due to observed cell loss, Wnt inhibition was performed using 4 µM ZAV939 in RPMI/B-27 minus insulin for 24–48 h. Subsequently, cultures were maintained in RPMI 1640 supplemented with B-27 with insulin, with medium changes every 24–48 h. Spontaneous beating was observed between days 7–10, consistent with CM differentiation. Thereafter, cells were maintained with medium changes every 1–2 days. Metabolic selection of hiPSC-CMs was performed using RPMI 1640 without glucose, supplemented with B-27 with insulin and 4 µM L-lactate [23]. Cells were used for experiments once stable contractility and CM-like morphology were established.

### Calcium Assay

Intracellular calcium handling was evaluated using a fluorescent calcium-sensitive dye, Fluo-4 AM (2–5 µM final concentration). Engineered heart tissue or CMs were incubated with dye for 20–30 min at 37 °C, followed by a 10-min de-esterification period. Samples were transferred to Tyrode’s or HBSS buffer for imaging. Calcium flux was recorded using high-speed fluorescence imaging, and peak amplitude, rise time, and decay kinetics were quantified using ImageJ. All measurements were performed at 37 °C to maintain physiologic conditions.

### Transmission Electron Microscopy

Ultrastructural analysis of hiPSC-CMs was performed using a Hitachi HT7800 transmission electron microscope housed in the Cincinnati Children’s Hospital Microscopy Core. Samples were fixed in 2.5% glutaraldehyde in 0.1 M cacodylate buffer at 4 °C overnight, washed thoroughly, and post-fixed with 1% osmium tetroxide for 1 h at room temperature. Tissues were then dehydrated through a graded ethanol series and embedded in epoxy resin. Ultrathin Sections. (70–90 nm) were cut using an EM UC7 ultramicrotome, collected onto copper grids, and stained sequentially with uranyl acetate and lead citrate. Images were acquired at 80–120 kV accelerating voltage using standard biological imaging settings to capture sarcomeric structure, mitochondrial morphology, and nuclear features.

### Scanning Electron Microscopy

Microparticle morphology was analyzed using a Hitachi SU8010 field-emission scanning electron microscope at the Cincinnati Children’s Hospital Microscopy Core. Lyophilized PLGA microparticles were mounted on aluminum stubs using carbon adhesion tabs and sputter-coated with a 5–10 nm layer of gold–palladium to ensure conductivity. Samples were imaged at accelerating voltages between 5 and 10 kV, with magnifications selected to resolve surface topography and pore structure. Images were captured under high-vacuum mode using secondary electron detection.

### RNA Sequencing Analysis

Total RNA was extracted from engineered tissues using a silica column-based purification kit, with on-column DNase digestion. RNA concentration and purity were assessed by NanoDrop spectrophotometry, and integrity was confirmed via Bioanalyzer (RIN ≥ 7 required for library preparation). Approximately 200 ng of total RNA for each sample was prepared for conventional sequencing libraries using the TruSeq RNA Library Prep Kit (Illumina) as per the manufacturer’s instructions, with mRNA enriched via poly-A-selection using oligoDT beads. RNA was then thermally fragmented with random priming, converted to cDNA, and PCR amplified. The libraries were sequenced using the Illumina HiSeq 2500 platform with 100-nucleotide paired-end reads as per the manufacturer’s instructions. Raw reads were quality-filtered and aligned to the human reference genome using Salmon [24]. Differential gene expression analysis was performed using DESeq2 [25], with genes considered significantly regulated based on adjusted *p* < 0.05 and fold-change > 2 or < 0.5 thresholds. Enrichment analyses, including GO term analysis and GSEA, were conducted using clusterProfiler [26] to identify functional pathways associated with observed transcriptional changes.

### TUNEL Staining

Apoptotic cells were quantified using TUNEL staining following the manufacturer’s kit instructions. Engineered tissues or cryosectioned heart samples were fixed in 4% paraformaldehyde, permeabilized with 0.1% Triton X-100, and incubated with TUNEL reaction mixture for 1 h at 37 °C. After washing, samples were counterstained with DAPI, mounted, and imaged using fluorescence microscopy. The percentage of TUNEL-positive nuclei was calculated relative to total nuclei within each field.

### Mitochondrial Imaging Assays

Mitochondrial activity was assessed using MitoTracker Red CMXRos (100 nM). Live cells or engineered tissues were incubated with the dye at the recommended working concentration for 20–30 min at 37 °C, followed by washing with warm PBS to remove unbound dye, and imaged immediately to avoid photobleaching. Confocal microscopy was used to capture mitochondrial distribution and fluorescence intensity. Signal quantification was performed using ImageJ by thresholding and averaging intensity per unit area.

Mitochondrial membrane potential (ΔΨm) was assessed using the JC-1 dye (abcam, ab113850). hiPSC-CMs were incubated with JC-1 working solution for 10 min at 37 °C, consistent with protocols for adherent cells. Following incubation, cells were gently washed with dilution buffer to remove excess dye and immediately imaged under fluorescence microscopy. Mitochondrial depolarization was quantified by measuring the ratio of red J-aggregate fluorescence (590 ± 17.5 nm) to green monomer fluorescence (530 ± 15 nm) using ImageJ, with reduced red/green ratios indicating loss of ΔΨm.

### Fabrication of Hyaluronic Acid-based 3D Oxygen-releasing Engineered Heart Tissue

To generate three-dimensional oxygen-releasing engineered heart tissue (OR-EHT), purified hiPSC-derived cardiomyocytes (hiPSC-CMs) were encapsulated within a thiol-modified hyaluronic acid-based hydrogel matrix (HyStem®, Advanced BioMatrix) in the presence or absence of oxygen-generating particles (OGPs). Briefly, the HyStem® Hydrogel Kit (Advanced BioMatrix) was utilized to encapsulate hiPSC-CMs and OGPs prior to transplantation. In accordance with the manufacturer’s instructions, hiPSC-CMs were resuspended in Glycosil (HA-based precursor solution) at a final concentration of ~ 1 × 10^6^ cells/mL, with OGPs added at 2 mg/mL. The mixture was gently pipetted to achieve initial dispersion, incubated for 5 min, and then pipetted again to ensure uniform distribution of cells and particles. Hydrogel matrix crosslinking was initiated by adding Extralink-Lite to the Glycosil solution at a 1:4 volume ratio, yielding a three-dimensional matrix. The constructs were incubated under standard culture conditions for ~ 12 h in standard CM medium to allow gel stabilization and formation of the “engineered heart tissue”. These patch-like hydrogel-based OR-EHTs were collected and subsequently applied directly to the epicardial surface of infarcted hearts for in vivo studies.

### Mouse MI Model and Transplantation

All research protocols conformed to the Guidelines for the Care and Use of Laboratory Animals published by the National Institutes of Health (National Academies Press, eighth edition, 2011). Immunodeficient nude mice (Stock #002019) and RFP-overexpressing mice (Stock #005884) were obtained from The Jackson Laboratory and subsequently crossed and inbred to generate RFP-positive nude mice. Genotyping was confirmed by standard PCR according to the manufacturer’s protocols. MI was induced in adult mice (8–10 weeks old) by permanent ligation of the left anterior descending (LAD) coronary artery under sterile surgical conditions. Animals were anesthetized with isoflurane (1–2%), intubated, and mechanically ventilated. A left thoracotomy was performed, and the LAD was ligated 1–2 mm below the tip of the left auricle using an 8–0 suture. Successful infarction was confirmed visually by myocardial blanching of the anterior wall. For EHT transplantation, a total volume of 1 mL (approximately 1 × 10^6^ hiPSC-CMs within 3D hydrogel collected from a 3-cm dish) was applied to the peri-infarct region. Animals were monitored postoperatively and provided analgesia (buprenorphine) following institutional animal care guidelines.

### Masson Trichrome Staining

Heart tissues were fixed in 4% paraformaldehyde, paraffin-embedded, and sectioned at 5–7 µm thickness prior to Masson trichrome staining to assess fibrosis and scar formation. Sections were processed through standard staining steps to visualize collagen (blue), muscle (red), and nuclei (black). Slides were imaged using bright-field microscopy. Fibrotic area and infarct size were quantified from scanned images using ImageJ.

### Echocardiography

Cardiac function was evaluated using transthoracic echocardiography under light isoflurane anesthesia (0.5–1%). M-mode and B-mode images were obtained in parasternal long-axis and short-axis views. Left ventricular ejection fraction, fractional shortening, and wall thickness measurements were quantified using manufacturer-provided software. All imaging was performed by an operator blinded to treatment groups.

### Immunohistochemistry

Tissue sections were deparaffinized, rehydrated, and subjected to antigen retrieval (pH 6.0, 95 °C for 10 min) prior to blocking in 5% serum. Sections were incubated with primary antibodies overnight at 4 °C, washed, followed by species-appropriate fluorescent secondary antibodies for 1 h at room temperature. Primary antibodies used were anti-cardiac troponin T (cTnT) (rabbit, proteintech, 15513–1-AP); anti-α-actinin (rabbit, proteintech, 14221–1-AP); anti-HIF-1α (rabbit, Cell Signaling, 36169); DCFDA Cellular ROS Assay Kit (abcam, ab1113851). Secondary antibodies used were: Alexa Fluor-conjugated (rabbit, invitrogen, a11008). Nuclei were counterstained with DAPI (invitrogen, D21490), and slides were mounted for confocal imaging. Marker expression, cell engraftment, and tissue remodeling were quantified by standardized image analysis.

### Statistical Analysis

All experiments were independently repeated at least three times and yielded consistent results. The number of biological or technical replicates (n) for each experiment is indicated in the corresponding figure legends. Data are expressed as mean ± SD unless otherwise noted. Statistical analyses were performed using GraphPad Prism. Comparisons between two groups used unpaired two-tailed Student’s t-tests, while multiple-group comparisons employed one-way or two-way ANOVA with Tukey’s post-hoc test. Non-parametric tests were applied where appropriate. A p-value < 0.05 was considered statistically significant. Sample sizes, statistical tests, and replication information are noted in the corresponding figure legends.

## Results

### Fabrication and Characterization of Antioxidant-integrated OGPs

**Fig. 1:**
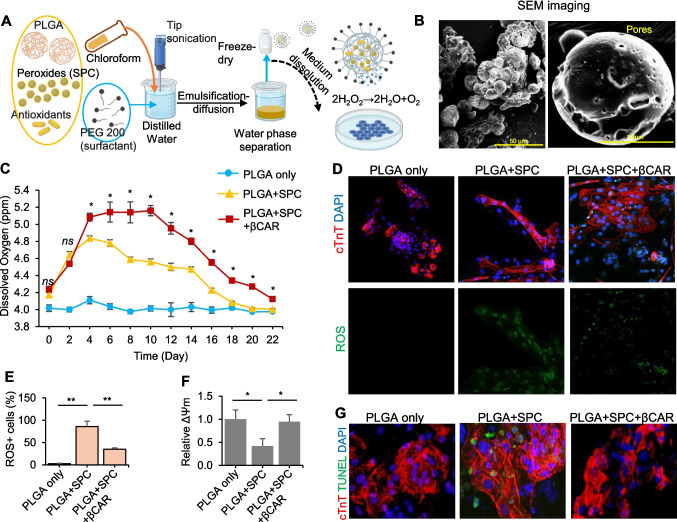
Characterization of oxygen-generating particles. (**A**) Schematic illustration of the fabrication of oxygen-generating particles composed of PLGA, SPC, and βCAR. (**B**) Scanning electron microscopy (SEM) image of PLGA microparticles. (**C**) Dynamics of dissolved oxygen (O₂) release from PLGA microparticles with or without SPC or βCAR. *n* = 5 per group. Compared with PLGA + SPC: *p* < 0.05; ns, p > 0.05. (**D**) Representative ROS imaging of hiPSC-CMs expressing cTnT following treatment with different particles for 24 h. (**E**) Quantification of intracellular ROS levels in hiPSC-CMs after 24 h of treatment with different particles. *n* = 5 per group. **p < 0.01. (**F**) Quantification of mitochondrial membrane potential in hiPSC-CMs after 24 h of treatment with different particles. *n* = 5 per group. *p < 0.05. (**G**) Representative TUNEL staining of cTnT-positive hiPSC-CMs after treatment with different particles for 24 h

Oxygen-generating particles (OGPs) were fabricated by encapsulating sodium percarbonate and the antioxidant βCAR within poly(lactic-co-glycolic acid) (PLGA) microparticles via a sonication-assisted emulsification-diffusion method (Fig. 1A). Hydrolytic degradation of PLGA enables gradual SPC release and decomposition to hydrogen peroxide (H_2_O_2_), which subsequently yields molecular oxygen (O_2_). Scanning electron microscopy (SEM) showed microparticles of ~ 10 μm diameter with a porous surface morphology consistent with controlled release (Fig. 1B). Dissolved-oxygen measurements in sealed vials using a Clark-type oxygen probe demonstrated a gradual, sustained increase in O_2_ concentration over 22 days. Inclusion of βCAR modestly increased total oxygen generated compared with SPC-only particles (Fig. 1C).

Because peroxide-based O_2_ generation can transiently elevate ROS, oxidative stress was assessed in hiPSC-CMs. The hiPSC-CMs were generated using established Wnt activation/inhibition protocols [27] and maintained in a lactate-based culture system [23] (Supplementary Fig. 1A). Successful CM differentiation was further confirmed by immunostaining for the canonical CM marker cTnT, which demonstrated the characteristic striated CM structure (Supplementary Fig. 1B). Stable hiPSC-CMs were collected for microparticle studies after spontaneous beating was observed between days 7 and 10 of differentiation. Using this approach, the CM differentiation efficiency consistently exceeded 90%, and treatment with the oxygen-generating particles did not affect the differentiation process (Supplementary Fig. 1C). After 24 h of exposure, DCFDA staining revealed significantly increased ROS levels in SPC-PLGA-treated cells, whereas the βCAR-containing formulation markedly attenuated this effect (Fig. 1D and E). Consistent with reduced oxidative stress, JC-1 staining showed diminished ΔΨm with SPC-PLGA that was largely preserved when βCAR was incorporated (Fig. 1F). Furthermore, TUNEL staining further demonstrated substantial apoptosis following SPC-PLGA treatment, while SPC-PLGA-βCAR significantly reduced cell death (Fig. 1G). Collectively, these data indicate that antioxidant-integrated OGPs (SPC-PLGA-βCAR) provide sustained oxygen release while minimizing oxidative toxicity and preserving mitochondrial integrity, thereby creating a cytoprotective microenvironment for hiPSC-CMs.

### OGPs Accelerate Functional Development of hiPSC-CMs

**Fig. 2:**
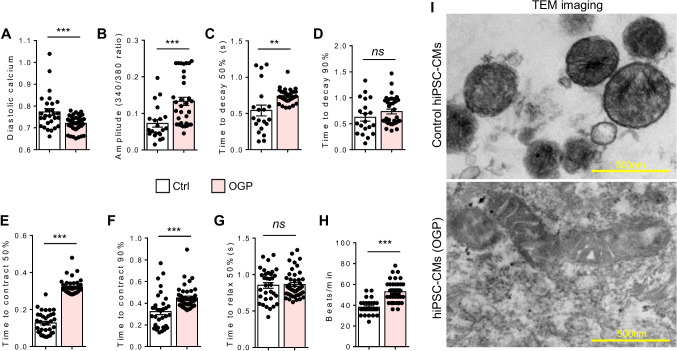
Effects of particles on the contractile function and mitochondrial ultrastructure of hiPSC-CMs. (**A**-**H**) Contractile parameters of hiPSC-CMs treated with or without oxygen-generating particles (OGPs) for 24 h were analyzed using the IonOptix contractility system at a stimulation frequency of 0.5 Hz. Measurements were obtained from *n* = 22–40 cells per group across three independent experiments. Statistical significance: *p* < 0.05, *p < 0.01, **p < 0.001; ns, not significant. (I) Representative transmission electron microscopy (TEM) images showing mitochondrial ultrastructure in hiPSC-CMs after 24 h exposure to the indicated particles

To determine whether OGPs modulate excitation–contraction coupling, intracellular Ca^2+^ transients were quantified in hiPSC-CMs. Baseline diastolic Ca^2+^ levels were significantly lower in OGP-treated cells relative to controls (Fig. 2A), consistent with improved resting Ca^2+^ clearance. Peak systolic Ca^2+^ amplitude was significantly increased with OGP treatment (Fig. 2B), indicating enhanced Ca^2+^ release capacity. The time required for Ca^2+^ decay to reach 50% of peak amplitude (T50) was significantly prolonged in OGP-treated cells (Fig. 2C), whereas the time to 90% decay (T90) was unchanged (Fig. 2D), suggesting selective slowing of early-phase Ca^2+^ reuptake without affecting late relaxation. Mechanical analysis showed a corresponding prolongation in the time to 50% and 90% contraction (Fig. 2E-F), while the time to 50% relaxation was unchanged (Fig. 2G). Spontaneous beating frequency was markedly increased in the OGP group (Fig. 2H), reflecting enhanced automaticity. Transmission electron microscopy (TEM) revealed elongated mitochondria with more developed cristae in OGP-treated hiPSC-CMs, whereas control cells exhibited smaller, rounder mitochondria with less-organized cristae (Fig. 2I). These features are consistent with improved early mitochondrial structural maturation. Together, these findings show that OGP treatment modulates Ca^2+^-cycling dynamics, prolongs contraction kinetics, increases spontaneous activity, and enhances mitochondrial structural maturation in hiPSC-CMs.

### OGPs Promote Transcriptional Programs Associated With Cardiomyocyte Maturation

**Fig. 3:**
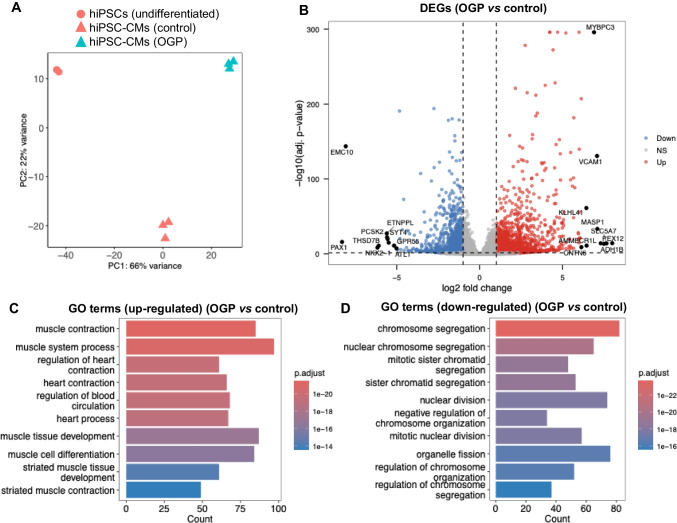
Transcriptomic profiling of hiPSC-CMs in response to oxygen-generating particles. (**A**) Principal component analysis (PCA) of global transcriptomic profiles in the tested cell groups. (**B**) Volcano plot showing differentially expressed genes in hiPSC-CMs treated with oxygen-generating particles (OGPs) compared with untreated controls. (**C**-**D**) Top Gene Ontology (GO) biological processes enriched among significantly upregulated (**C**) and downregulated (**D**) genes in hiPSC-CMs treated with OGPs for 24 h relative to the control group

To characterize molecular consequences of OGP exposure, RNA-seq was performed on hiPSC-CMs treated with OGPs or vehicle for 24 h. Principal component analysis (PCA) of the 500 most variable genes showed clear separation between groups, indicating high biological reproducibility and distinct transcriptional profiles (Fig. 3A). Differential expression analysis identified 1,132 upregulated and 742 downregulated genes in OGP-treated cells versus controls (Fig. 3B). Among upregulated genes, *MYBPC3*, a key regulator of sarcomeric organization and contractile function [28], was significantly increased following OGP treatment. Gene Ontology (GO) enrichment analysis demonstrated that upregulated genes were predominantly associated with heart contraction, muscle system processes, and muscle development (Fig. 3C), whereas downregulated genes were enriched for cell cycle-related pathways, including chromosome segregation and mitotic nuclear division (Fig. 3D). These transcriptomic signatures suggest that OGP treatment shifts hiPSC-CMs away from proliferative states toward a more mature, contractile molecular phenotype.

### OGPs Enhance the Resistance of hiPSC-CMs to Hypoxic Stress

**Fig. 4:**
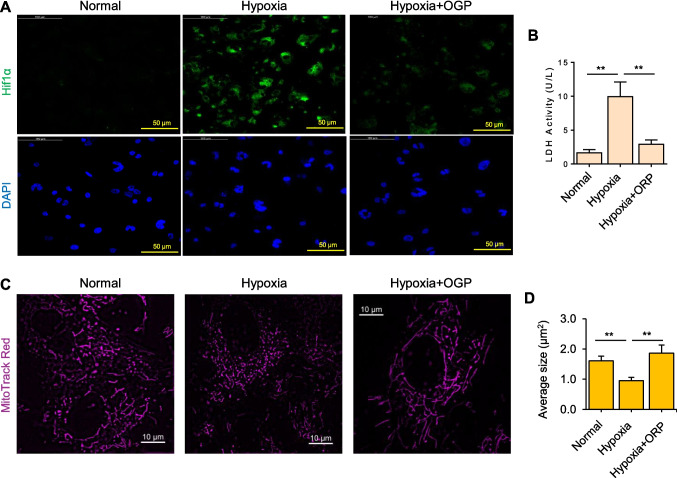
Effects of hypoxia and particle treatment on hiPSC-CMs. (**A**) Representative immunofluorescence images showing HIF1α expression in hiPSC-CMs treated with different conditions under hypoxia for 24 h (normoxia as the control). (**B**) Lactate dehydrogenase (LDH) activity assay measuring cell injury in hiPSC-CMs under hypoxic conditions with different treatments for 24 h (normoxia as the control). *n* = 5 per group. **p < 0.01. (**C**-**D**) Representative MitoTracker Red staining images (**C**) and quantification of mitochondrial area (**D**) in hiPSC-CMs after 24 h exposure to hypoxia with the indicated treatments (normoxia as the control)

To test whether OGPs improve resilience under hypoxia, hiPSC-CMs were exposed to 24 h of low O_2_ after OGP pretreatment. Hypoxia markedly increased HIF-1α expression, whereas this induction was substantially attenuated with OGP pretreatment (Fig. 4A). Consistent with reduced injury, LDH activity was significantly elevated under hypoxia but decreased toward baseline levels in OGP-treated cells (Fig. 4B). Mitochondrial morphology was assessed using MitoTracker Red staining (Fig. 4C). Under hypoxia, hiPSC-CMs exhibited perinuclear mitochondrial clustering accompanied by fragmented and condensed mitochondria, features indicative of mitochondrial distress. In contrast, OGP-pretreated cells maintained elongated, interconnected mitochondrial networks resembling normoxia. Quantitative analysis confirmed significantly greater mitochondrial area in OGP-pretreated cells compared with hypoxic controls (Fig. 4C-D). These findings indicate that OGPs mitigate hypoxia-induced metabolic and structural injury in hiPSC-CMs by preserving mitochondrial integrity and reducing cellular stress responses.

### Oxygen-generating Particles Activated Transcriptional Programs Counteracting Hypoxia-Associated Stress

**Fig. 5:**
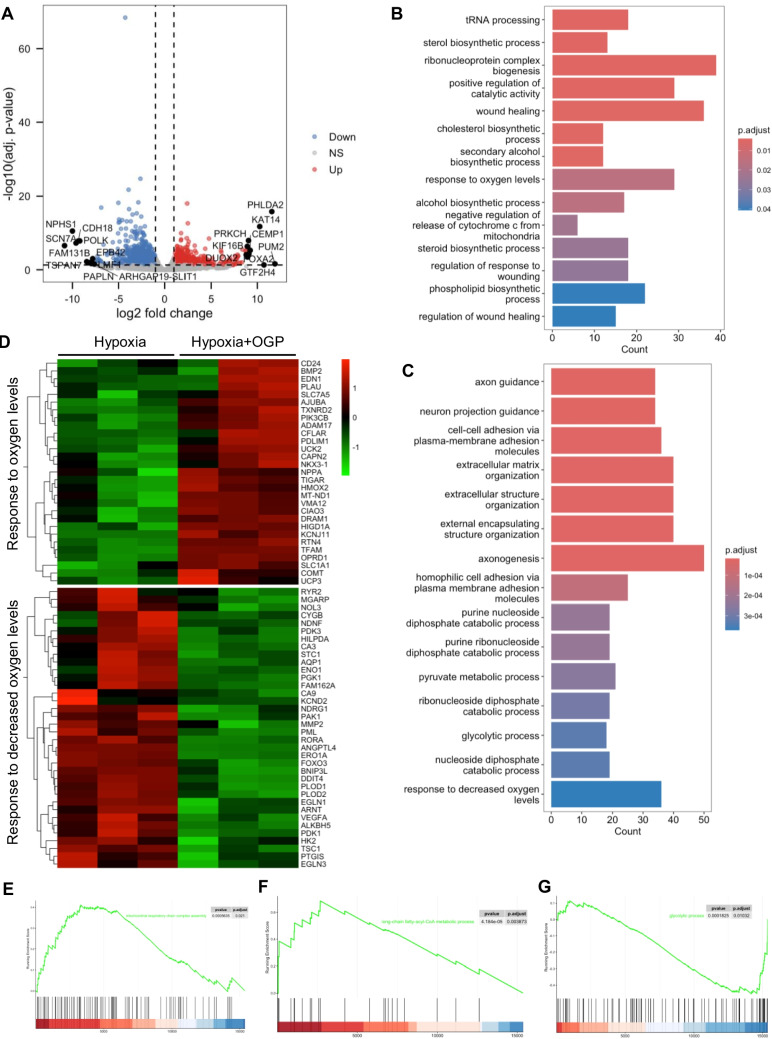
Transcriptomic responses of hiPSC-CMs to hypoxia and oxygen-generating particle treatment. (**A**) Volcano plot illustrating differentially expressed genes in hiPSC-CMs treated with oxygen-generating particles (OGPs) compared with untreated controls under hypoxic conditions for 24 h. (**B**-**C**) Top Gene Ontology (GO) biological processes enriched among significantly upregulated (**B**) and downregulated (**C**) genes in OGP-treated hiPSC-CMs relative to controls under hypoxia for 24 h. (**D**) Heatmap of gene expression related to the GO terms of interest across hiPSC-CMs with or without OGPs under hypoxia. (**E**–**G**) Gene set enrichment analysis (GSEA) identifying significantly activated (E and E) or repressed (G) biological pathways in hiPSC-CMs following OGP treatment under hypoxic conditions for 24 h

To examine how OGPs influence the transcriptional response to hypoxia, RNA-seq was performed on hiPSC-CMs exposed to hypoxia with or without OGP pretreatment. Differential expression analysis identified 751 upregulated and 979 downregulated genes in OGP-treated cells relative to hypoxic controls (Fig. 5A). Among the upregulated genes, *DUOX2*, an oxidase involved in redox signaling, was significantly increased following OGP treatment. GO enrichment analysis demonstrated a shift in oxygen-responsive programs: upregulated genes were enriched for response to oxygen levels (Fig. 5B), whereas downregulated genes were enriched for response to decreased oxygen levels (Fig. 5C), consistent with reduced hypoxic stress. Heatmap visualization (Fig. 5D) highlighted induction of factors linked to mitochondrial homeostasis and oxidative-stress adaptation including *TFAM*, *MT-ND1*, *TXNRD2*, *HIGD1A*, *UCP3*, *HMOX2*, and *DRAM1*, along with signaling and remodeling genes such as *EDN1*, *ADAM17*, *PLAU*, *CAPN2*, and *BMP2*. Conversely, hypoxic control hiPSC-CMs exhibited stronger activation of canonical HIF-dependent hypoxia programs (*CA9*, *VEGFA*, *EGLN1*, *EGLN3*, *ARNT*, *DDIT4*, *HILPDA*, *ANGPTL4*, *NDRG1*, *STC1*), glycolytic metabolism (*HK2*, *PGK1*, *ENO1*, *PDK1*, *PDK3*) and stress/mitophagy genes (*BNIP3L*, *FAM162A*). Gene-set enrichment analysis (GSEA) further showed enrichment of mitochondrial respiratory-chain assembly and long-chain fatty-acyl-CoA metabolic processes in OGP-treated cells (Fig. 5E and F), whereas glycolysis was enriched in hypoxic controls (Fig. 5G). These patterns indicate that OGP pretreatment partially alleviates hypoxia in hiPSC-CMs, reducing reliance on canonical hypoxia-adaptation pathways while promoting mitochondrial and redox programs consistent with improved oxygen availability.

### Generation of OR-EHT for MI Repair

**Fig. 6:**
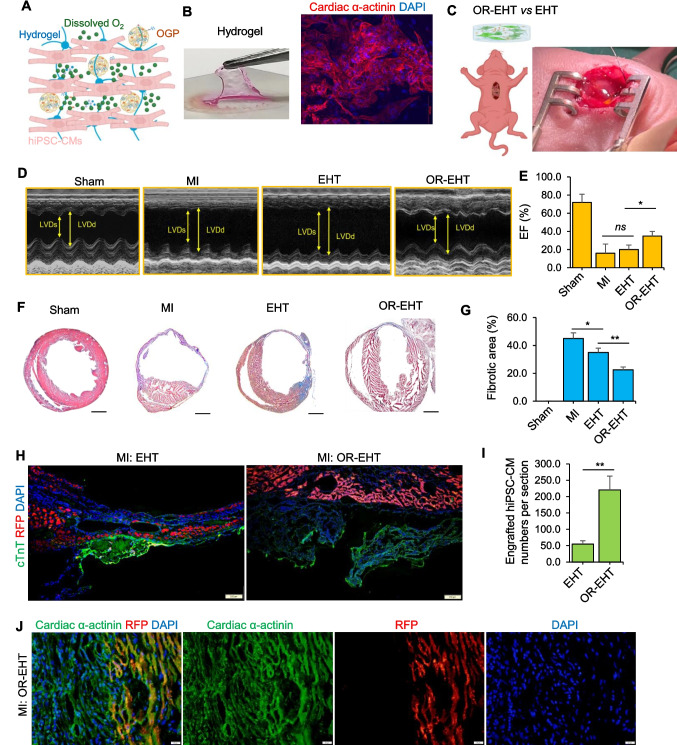
Functional and histological assessment of infarcted hearts treated with engineered heart tissue. (**A**) Schematic illustration of the fabrication of oxygen-releasing engineered heart tissue (OR-EHT) composed of hiPSC-derived cardiomyocytes and oxygen-generating particles encapsulated within a 3D hydrogel scaffold. (**B**) Hydrogel constructs and α-actinin immunostaining showing cardiomyocyte organization. (**C**) Implantation of hydrogel patches onto the epicardial surface of infarcted hearts. (**D**) Representative M-mode echocardiography images from different mouse groups 6 weeks after surgery. (**E**) Quantification of left ventricular ejection fraction (EF) at 6 weeks post-MI (*n* = 7 per group). *p* < 0.05; ns, not significant. (**F**-**G**) Masson’s trichrome staining of heart sections and quantification of infarct and fibrotic areas at 6 weeks post-MI (*n* = 5 per group). * *p* < 0.05; ** *p* < 0.01. Scale bars, 1 mm. (**H**) Representative images of cTnT-positive EHT grafts within RFP-expressing infarcted myocardium 6 weeks after implantation. Scale bars, 200 µm. (**I**) Quantification of cTnT-positive cardiomyocyte retention in MI mice (*n* = 5 per group). ** *p* < 0.01. (**J**) Representative images of OR-EHT grafts identified by sarcomeric α-actinin staining in the infarct border zone 6 weeks after implantation. Scale bars, 20 µm

To enable localized oxygen delivery and support tissue organization, purified hiPSC-CMs and OGPs were encapsulated within a thiol-modified hyaluronan hydrogel matrix, forming a three-dimensional (3D) oxygen-releasing engineered heart tissue (OR-EHT) (Fig. 6A-B). This 3D scaffold that mimics aspects of the native cardiac extracellular matrix and allows uniform spatial distribution of cells and microparticles. The patch-like hydrogel-based “engineered heart tissue” (EHT) can support cell viability, structural support, organization, and function, as well as a localized microenvironment for sustained oxygen delivery. Therapeutic efficacy was tested in an immunodeficient mouse MI model (RFP-transgenic recipients). Following permanent LAD ligation, hydrogel patches were applied to the epicardial surface over the infarct. At 6 weeks post-MI, echocardiography showed that OR-EHT significantly improved cardiac function compared with the MI-only and EHT-only groups, with reduced left ventricular end-diastolic and end-systolic diameters (LVDd and LVDs, respectively). The hydrogel matrix also provided a supportive viscoelastic microenvironment for CM expansion and organization, as evidenced by the expression of the sarcomeric protein α-actinin (Fig. 6B). Purified hiPSC-CMs were encapsulated with OGPs in the hydrogel to generate oxygen-releasing engineered heart tissue (OR-EHT). The therapeutic potential of OR-EHT was tested in an MI mouse model using RFP-transgenic nude mice as recipients (Fig. 6C). Following LAD ligation, hydrogel patches were applied to the epicardial surface to cover the infarcted myocardium. Echocardiographic analysis demonstrated that OR-EHT treatment significantly improved cardiac function, with reduced left ventricular end-diastolic diameter (LVDd) and left ventricular end-systolic diameter (LVDs) and increased ejection fraction (EF) compared with MI-only or EHT-treated mice at 6 weeks post-MI (Fig. 6D-E). Histological analysis using Masson trichrome staining further revealed a reduced infarct/fibrotic size in OR-EHT-treated hearts compared with the EHT group (Fig. 6F-G). Using RFP-expressing recipient mice to distinguish host tissue, we observed significantly greater engraftment of cTnT-positive hiPSC-CMs in the infarct region following OR-EHT transplantation (Fig. 6H-I). Additionally, transplanted α-actinin-positive hiPSC-CMs were found to integrate with host CMs in the infarct border zone (Fig. 6J), indicating potential structural integration and functional contribution to myocardial repair.

## Discussion

One of the primary challenges limiting the therapeutic efficacy of hiPSC-CM transplantation for MI is the hostile microenvironment of the infarcted myocardium. Severe hypoxia, elevated ROS, inflammatory signaling, and impaired vascular supply collectively cause rapid donor-cell death within the first few days after transplantation [29]. In this study, we addressed this limitation through the development of an oxygen-generating platform capable of sustained oxygen release while simultaneously buffering ROS. By encapsulating SPC with the antioxidant βCAR in PLGA microparticles and integrating them into a hyaluronic acid hydrogel, we established a controlled strategy to alleviate hypoxia and limit oxidative stress in EHT. Our findings demonstrate that controlled oxygen delivery, when coupled with antioxidant stabilization, preserves mitochondrial integrity, normalizes metabolic programming, improves excitation–contraction coupling, and enhances graft retention in vivo.

Previous studies have explored oxygen-generating biomaterials to improve cell survival in ischemic environments. Peroxide-based systems have shown the ability to transiently increase oxygen availability and improve survival of various transplanted cell types [10, 30]. For example, peroxide-releasing microspheres embedded in hydrogels have improved the survival of cardiac progenitor cells under hypoxia, and oxygen-releasing injectable systems have enhanced angiogenesis and reduced fibrosis after MI [19, 31]. More recently, Mandal et al*.* demonstrated that PLGA/CaO₂ microparticles improved CM metabolic activity under hypoxia by suppressing HIF-1α activation, although oxygen generation was short-lived [32]. The two major limitations of these systems, rapid oxygen bursts followed by depletion and production of ROS intermediates during peroxide decomposition, have limited their translational potential [12]. In contrast, our platform provides multi-week oxygen release and incorporates an intrinsic antioxidant component to mitigate ROS, addressing both drawbacks.

Two design features distinguish the present OGP system from earlier approaches. First, the integration of βCAR provides an embedded antioxidant buffer to counteract ROS generated during sodium percarbonate decomposition [33]. While oxygen delivery can support cell survival, excessive oxidative stress is detrimental to CM viability and mitochondrial function [34]. By incorporating an antioxidant component within the particle formulation, our platform simultaneously increases oxygen availability while maintaining redox balance. A similar conceptual approach has been explored in antioxidant-releasing cardiac patches designed to mitigate oxidative damage following MI; however, these systems were typically developed for a cell-free exosome-delivering patch rather than a CM-supportive engineered tissue [35]. Co-encapsulation of SPC with βCAR therefore maintains redox balance during oxygen release. Second, our data demonstrates that oxygen supplementation affects not only acute cytoprotection but also promotes upregulation of broader transcriptional and functional development programs in hiPSC-CMs, including contractile gene expression, mitochondrial remodeling, and improved calcium handling.

OGP treatment enhanced several functional parameters associated with early/partial CM maturation. Calcium imaging revealed increased systolic calcium amplitude and reduced diastolic calcium levels, consistent with improved calcium handling and excitation–contraction coupling in mature CMs [36, 37]. However, the observed prolongation of early calcium decay suggests that OGP treatment may differentially influence components of calcium reuptake, such as SERCA activity or buffering capacity, rather than uniformly accelerating calcium clearance as seen in fully mature adult CMs [38].

Ultrastructural analysis further demonstrated elongated mitochondria with well-defined cristae in OGP-treated CMs, depicting enhanced mitochondrial structural maturation [39, 40]. In immature CMs or metabolically stressed CMs, mitochondria frequently exhibit fragmented morphologies associated with impaired respiratory complex organization and reduced oxidative efficiency [41, 42]. Although mitochondrial morphology alone is not sufficient to establish full metabolic maturation, the structural remodeling observed here is supported by transcriptomic and functional data, indicating that OGP exposure contributes to a shift towards a more energetically mature phenotype. Nevertheless, definitive demonstration of full cardiomyocyte maturation would require additional electrophysiological and structural analyses, including assessment of action potential properties, conduction behavior, sarcomere ultrastructural organization, and T-tubule development.

Beyond functional development, OGP treatment modulated the hypoxic response itself. Under normoxic conditions, the transcription factor HIF-1α is constitutively synthesized but rapidly hydroxylated by prolyl hydroxylase domain enzymes (PHDs), ubiquitinated by the von Hippel-Lindau E3 ligase complex, and targeted for proteasomal degradation [43]. Hypoxia stabilizes HIF-1α, driving the expression of genes involved in glycolysis, angiogenesis, and metabolic reprogramming toward anaerobic ATP production [43–45]. The hypoxic exposure in our system induced robust HIF-1α accumulation and elevated LDH release, consistent with metabolic stress and cellular injury. However, these responses were markedly attenuated in OGP-treated CMs, indicating that localized oxygen release effectively mitigates the metabolic consequences of oxygen deprivation. Transcriptomic analysis further supported this interpretation, revealing coordinated suppression of canonical hypoxia-responsive genes, including *VEGFA*, *PDK1*, *HK2*, *EGLN3*, *ANGPTL4*, and *CA9*, [45–48] alongside reduced enrichment of GO categories associated with glycolysis and cellular responses to reduced oxygen tension [9]. Conversely, pathways associated with biosynthetic activity and cellular recovery, including ribonucleoprotein complex biogenesis, lipid-related processes, mitochondrial respiratory, metabolic pathways, and wound-healing/cellular repair programs, were enriched in the OGP condition. PDK1, a key HIF‑1α-induced kinase that inhibits pyruvate dehydrogenase (PDH) and forces cells into anaerobic lactate production. This metabolic switch is maladaptive in CMs, which requires oxidative phosphorylation to sustain contraction [41, 45]. By attenuating PDK1, OGPs would reactivate PDH and improve flux through the tricarboxylic acid (TCA) cycle – a shift toward oxidative phosphorylation that underlies the improved contractile and metabolic function we observed [43, 45]. These findings align with previous studies showing that localized oxygen delivery improves CM survival and metabolic function under hypoxia, but extend them by demonstrating broader reprogramming of metabolic and stress-response pathways [19, 31, 32]. However, recent work in the cardiac regeneration field has primarily reported improvements in short-term cell viability or reductions in HIF-1α signaling following oxygen delivery. Our transcriptomic data extend these observations by suggesting that sustained oxygen supplementation may influence the broader transcriptional and metabolic landscape of immature CMs, promoting a shift from hypoxia-adapted, glycolysis-dominant state toward mitochondrial metabolic competence. Collectively, these results support the emerging concept that oxygen-generating biomaterials can serve as active modulators of CM metabolic programming and maturation, which may have critical implications for improving the functional integration and long-term performance of transplanted hiPSC-CMs in ischemic myocardium.

These cellular and molecular improvements correspond with enhanced therapeutic outcomes in vivo. In the mouse MI model, transplantation of OR-EHT resulted in significant improvements in cardiac function, including increased ejection fraction and reduced ventricular dilation. Masson trichome staining revealed smaller infarct and fibrotic areas in OR-EHT-treated hearts, and significantly greater retention of transplanted hiPSC-CMs was observed. The most direct mechanism can be attributed to reduced early CM death, hypoxia-induced necrosis and apoptosis during the critical post-transplantation window. By locally sustaining oxygen availability, the OGP platform extends the survival of transplanted cells long enough for them to adapt to the ischemic environment. A second mechanism involves a shift in the metabolic state of the transplanted CMs. CMs that have been primed under OGP conditions to rely on oxidative phosphorylation rather than glycolysis may be better prepared to function in the oxygen-limited infarct border zone. A third possible suggested mechanism involves a potential paracrine effect of these metabolically competent, matured hiPSC-CMs that secrete a distinct profile of growth factors, cytokines, and extracellular vesicles compared to hypoxia-stressed cells. Some fractions of the observed reductions in fibrosis and improvements in cardiac function may be attributed to these indirect effects on host tissue remodeling. Nevertheless, these findings suggest that improved oxygen availability during the early post-transplantation period enhances graft survival, thereby enabling transplanted CMs to contribute more effectively to myocardial repair. Increased engraftment may also facilitate improved structural integration between donor and host myocardium, as suggested by the presence of α-actinin-positive CMs in the infarct border zone. However, while increased α-actinin-positive graft presence suggests structural integration, definitive evidence of electromechanical coupling and arrhythmia risk was not assessed and remains a critical barrier highlighted in large-animal studies [4, 5, 49]. Thus, while oxygen modulation clearly enhances graft persistence, its impact on functional integration and electrical stability requires further investigation.

While our findings highlight the potential of oxygen-releasing biomaterials for cardiac regeneration, several limitations should be acknowledged. First, although sustained oxygen release was observed for approximately three weeks, the actual oxygen tension experienced by cells within the engineered tissue and infarct microenvironment in vivo was not directly measured. Because oxygen diffusion in dense tissues is spatially heterogeneous, the relationship between released oxygen and biologically relevant oxygen exposure remains incompletely defined. Future studies using fiber-optic oxygen microsensors, phosphorescence lifetime imaging, and Electron Paramagnetic Resonance Oximetry [50, 51] will be important for mapping local oxygen gradients and establishing physiologically relevant oxygen dosing within engineered tissues and infarcted myocardium. Second, while βCAR reduced ROS generation in vitro, the long-term effects of sustained oxygen production and antioxidant release in vivo remain unclear. Excess oxygen delivery or degradation products from PLGA and peroxide intermediates could potentially influence redox signaling or inflammatory responses in the injured myocardium, necessitating further investigation of long-term tissue remodeling and safety. Third, the present study was performed in a murine MI model, which does not fully recapitulate the scale and physiological complexity of the human heart. Larger animal models will be necessary to determine whether sufficient oxygen delivery can be achieved in thicker tissues with greater metabolic demands and diffusion distances. Moreover, all experiments were performed using hiPSC-derived human cardiomyocytes; therefore, validation of these OGPs in primary adult human cardiomyocytes represents an important future direction to further enhance translational relevance. However, such studies remain challenging due to the limited availability and inter-sample variability of donor cardiac tissue specimens, the low yield of viable isolated adult human cardiomyocytes, and the lack of reliable methods for their isolation, long-term culture, and scalable expansion [52, 53]. In addition, the inherently non-proliferative nature of adult cardiomyocytes limits their sustained use in ex vivo experimental studies [52–54]. Adult human cardiomyocytes will also require further optimization of biomaterial and microparticle delivery conditions to support stable long-term functional analyses. Similar limitations have been widely recognized in cardiac tissue engineering and stem cell–based myocardial regeneration studies [6]. Finally, although improved CM survival and functional recovery were observed, the relative contributions of oxygen delivery versus antioxidant protection remain difficult to distinguish. Future studies using independently controlled oxygen-releasing or antioxidant systems could help clarify the mechanistic roles of each component.

Overall, these findings support the concept that microenvironmental oxygen regulation is a critical but underexplored parameter in cardiac regenerative therapy. From a translational perspective, the capacity to simultaneously support CM survival and promote metabolic maturation may be particularly important for future cardiac remuscularization strategies. Rather than focusing exclusively on cell source or biomaterial structure, future strategies may benefit from incorporating metabolic regulation as an integral component of engineered cardiac constructs. By coupling oxygen generation with antioxidant buffering and structural tissue support, the OR-EHT platform presented here represents one step toward integrating metabolic microenvironment engineering into stem cell-based cardiac repair.

## Supplementary Information

Below is the link to the electronic supplementary material.

Supplementary file1 (PDF 250 KB)

## Data Availability

All RNA-sequencing raw data of this study were deposited in the NCBI GEO (accession number GSE326195). Other materials or resources used in this study are available from the corresponding authors upon reasonable request.

## Code Availability

The codes used in this study are available from the corresponding authors upon reasonable request.
