# Supplementary material for: Oxygen-generating Microparticles Enhance Viability and Functionality of Human Pluripotent Stem Cell-derived Cardiomyocytes for Myocardial Infarction Therapy
Source: Stem Cell Rev Rep. 2026 May 27;22(6):2846–62. doi: 10.1007/s12015-026-11163-z (PMC13354643; doi:10.1007/s12015-026-11163-z)
Supplement: Supplementary file 1 — Supplementary file1 (PDF 250 KB) [file 12015_2026_11163_MOESM1_ESM.pdf]

Supplemental Figure 1

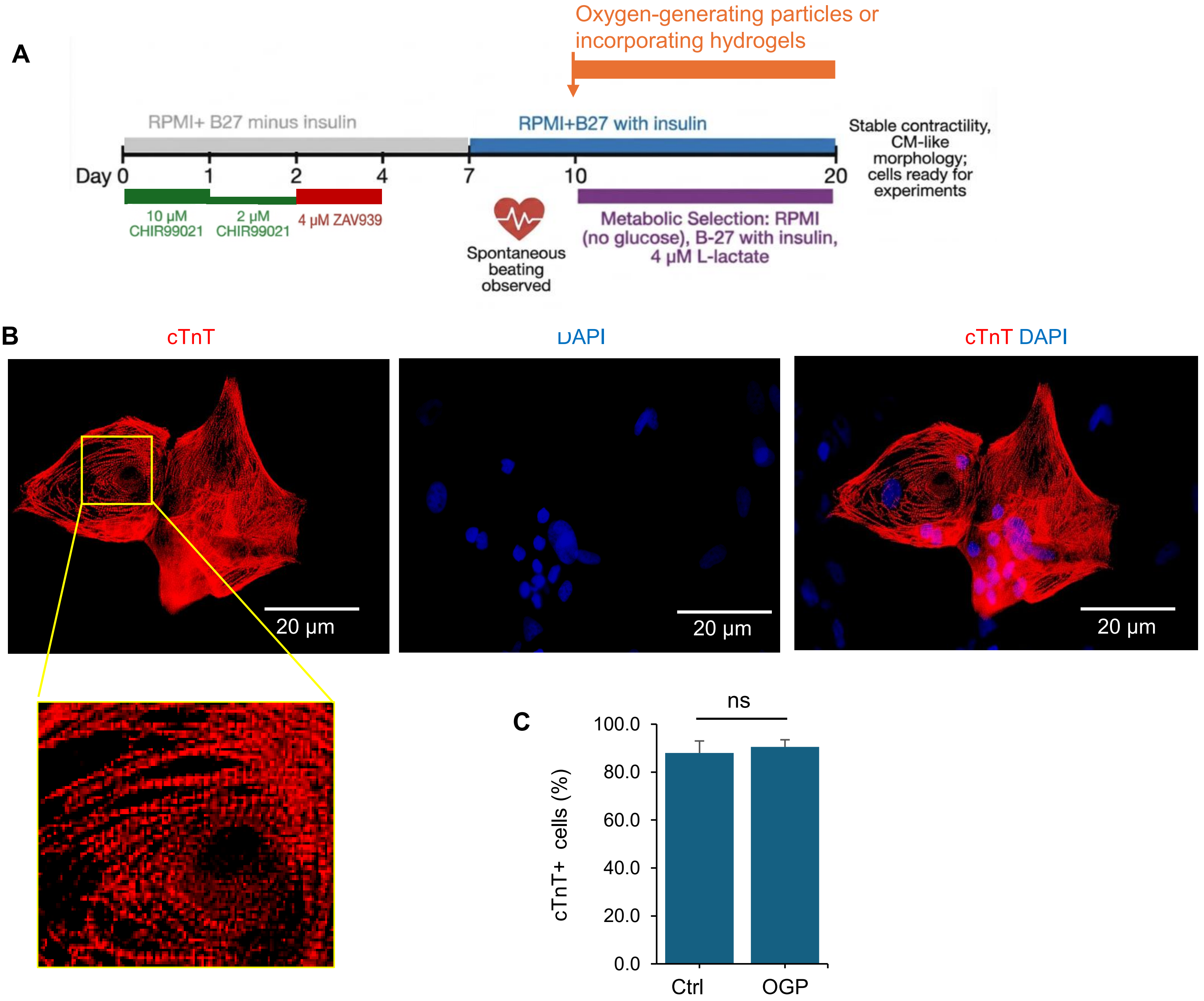

(A) Schematic overview of hiPSC-CM differentiation using established Wnt pathway activation/inhibition protocols and subsequent maintenance in a lactate-based culture medium. Oxygen-generating particles (OGPs) were introduced after spontaneous contractions were observed between days 7 and 10 of differentiation.

(B) Representative immunostaining of cardiac troponin T (cTnT) in hiPSC-CMs following 10 days of differentiation. A high-magnification image highlights the characteristic sarcomeric striations.

(C) Quantitative analysis of the proportion of cTnT-positive cells in hiPSC-CMs cultured with or without OGP treatment for 7 days. ns, no significance.
